# Combined serum levels of multiple proteins in tPA-BDNF pathway may aid the diagnosis of five mental disorders

**DOI:** 10.1038/s41598-017-06832-6

**Published:** 2017-07-31

**Authors:** Suzhen Chen, Haitang Jiang, Yang Liu, Zhenhua Hou, Yingying Yue, Yuqun Zhang, Fuying Zhao, Zhi Xu, Yinghui Li, Xiaodong Mou, Lei Li, Tianyu Wang, Jingjing Zhao, Chongyang Han, Yuxiu Sui, Ming Wang, Zhong Yang, Yan Lu, Yifeng Zhu, Jianhua Li, Xinhua Shen, Fei Sun, Qingsong Chen, Huanxin Chen, Yonggui Yuan

**Affiliations:** 1 Department of Psychosomatics and Psychiatry, ZhongDa Hospital, Medical School of Southeast University, Nanjing, 210009 P.R. China; 2 Institute of Psychosomatics, Medical School of Southeast University, Nanjing, 210009 P.R. China; 3 Institute of Neuropsychiatric, Brain Hospital, Nanjing Medical University, Nanjing, 210029 P.R. China; 4 Department of Psychiatry, Brain Hospital, Nanjing Medical University, Nanjing, 210029 P.R. China; 5 Department of Psychiatry, The Third People’s Hospital of Changshu, Suzhou, 215500 P.R. China; 6 Department of Psychiatry, The Fourth People’s Hospital of Zhangjiagang, Suzhou, 215600 P.R. China; 7 Department of Psychiatry, The Third People’s Hospital of Huzhou, Huzhou, 313000 P.R. China; 8 Department of Psychiatry, The Second People’s Hospital of Jingjiang, Taizhou, 214500 P.R. China; 9 Key Laboratory of Cognition and Personality, Ministry of Education; School of Psychology, Southwest University, Chongqing, 400175 P.R. China

## Abstract

Mental disorders are severe, disabling conditions with unknown etiology and are commonly misdiagnosed when clinical symptomology criteria are solely used. Our previous work indicated that combination of serum levels of multiple proteins in tissue plasminogen activator (tPA)-brain-derived neurotrophic factor (BDNF) pathway improved accuracy of diagnosis of major depressive disorder (MDD). Here, we measured serum levels of tPA, plasminogen activator inhibitor-1 (PAI-1), BDNF, precursor-BDNF (proBDNF), tropomyosin-related kinase B (TrkB) and neurotrophin receptor p75 (p75NTR) in patients with paranoid schizophrenia (SZ, n = 34), MDD (n = 30), bipolar mania (BM, n = 30), bipolar depression (BD, n = 22), panic disorder (PD, n = 30), and healthy controls (HCs, n = 30) by Enzyme-linked immunosorbent assay kits. We used receiver operating characteristic (ROC) curve to analyze diagnostic potential of these proteins. We found, compared with HCs, that serum tPA and proBDNF were lower in SZ, BM and BD; TrkB was lower in SZ and BD; and p75NTR was declined in SZ and BM. ROC analysis showed that combined serum level of tPA, PAI-1, BDNF, proBDNF, TrkB and p75NTR was better than any single protein in accuracy of diagnosis and differentiation, suggesting that the combination of multiple serum proteins levels in tPA-BDNF pathway may have a potential for a diagnostic panel in mental disorders.

## Introduction

Mental disorders are a major burden to patients, their families and society, affecting a large number of the population worldwide and leading to hundreds of billion dollars in health care and treatment costs and income loss. Moreover, more than one-third people experienced at least one mental disorder at some time in their life in most countries worldwide^1^. Reviews of surveys on prevalence indicate that anxiety disorders including generalized anxiety disorder and panic disorder (PD), mood disorders including major depressive disorder (MDD), bipolar mania (BM) and bipolar depression (BD), and schizophrenia (SZ) are common and have high lifetime prevalence^2–4^. A study conducted in four provinces of China suggested that the adjusted 1-month prevalence of total psychiatric disorders is 17.5%, and the highest is affective disorders (6.1%), followed by substance abuse disorders (5.9%), anxiety disorders (5.6%) and SZ (1.0%)^5^. Furthermore, researchers found that mental disorders are the world’s first leading cause of disability accounting for about 5.9% of the global disability adjusted life years (DALYs), much higher than that of neurological disorders (3.0%) and substance use disorders (1.5%) in 2010^6^. In the United States, the cost of SZ has exceeded 19 billion dollars, more than the total cost of all cancers in 1991^7^. Moreover, It has been even predicted that MDD would become the biggest contributor to the burden of healthcare by 2030^8^.

Accurate diagnosis of mental illness is important for the treatment. But until now, no biomarker has yet been approved to be used in diagnosis of any psychiatric disorder, and clinical criteria still remain the only diagnostic method^9^. However, there are limitations in the existing diagnostic criteria. It is highly subjective for clinicians to diagnose mental illness based solely on interviews. Moreover, some symptoms may not be unique to any single disorder. For example, delusions and hallucinations that occur in SZ are also present in mood disorders, especially in bipolar disorder^10^. Treatment may be delayed or inappropriate owing to incorrect diagnosis. Therefore, to develop an objective test that can help diagnosis is important and urgent.

The exact neuropathological basis of mental disorders remains unclear, and widely accepted objective diagnostic tools do not exist currently. Studies on potential biomarkers for mental disorders have intensively been conducted, concentrated mostly on the diagnostic values of genetics, proteomics, imaging and electrophysiology^7, 11^. Studies of quantitative and qualitative proteins in cerebrospinal fluid (CSF) and serum have indicated that they have a potential as diagnostic and discriminating tools for mental disorders^12–16^. However, there is currently no diagnostic tool based on biomarkers.

In the past decades, researchers investigated many different proteins in CSF, serum and plasma in patients with different psychiatric disorders. Initially, studies were mainly focused on a single protein and suggested that it was promising for diagnosing mental disorder. However, late studies showed that a single protein has a little diagnostic value among diverse populations because the results are not always consistent. Recently, studies found that combined different serum proteins would provide a much higher diagnostic value than a single protein^17–20^, which indicated that this kind of combination could potentially become a diagnostic tool.

In our previous study, we found that the serum tissue plasminogen activator (tPA)-brain-derived neurotrophic factor (BDNF) pathway was involved in the pathogenesis of MDD and the combination of serum tPA, BDNF, precursor-BDNF (proBDNF), tropomyosin-related kinase B (TrkB) and neurotrophin receptor p75 (p75NTR) in the pathway gave a better diagnostic power than any single protein^21^. There were also reports that peripheral proteins, such as BDNF, was altered in other common mental disorders, such as bipolar disorder and SZ^22, 23^. In present study, we have explored if the combination of serum concentrations of multiple proteins in tPA-BDNF pathway could aid diagnosis and differentiation of a variety of other mental disorders.

## Materials and Methods

### Subjects and clinical assessment

146 patients participated in our study, and were divided into five groups based on the diagnosis which was performed and confirmed by three levels of experienced clinical psychiatrists. Patients with SZ (n = 34, 15 males/19 females; average age: 34.35 ± 10.89 years), MDD (n = 30, 5 males/25 females; average age: 41.60 ± 12.43 years), BM (n = 30, 14 males/16 females; average age: 34.40 ± 11.63 years), BD (n = 22, 11 males/11 females; average age: 34.19 ± 13.67 years) and PD (n = 30, 15 males/15 females; average age: 35.50 ± 12.38 years), and 30 healthy controls (HCs, 16 males/14 females; average age: 35.60 ± 12.90 years) were included. Participants were recruited from Affiliated ZhongDa Hospital of Southeast University, Affiliated Brain Hospital of Nanjing Medical University, the Fourth People’s Hospital of Zhangjiagang, the Third People’s Hospital of Changshu, the Third People’s Hospital of Huzhou and the Second People’s Hospital of Jingjiang from April 2016 to August 2016. All patients met the following entry criteria: (1) inpatients or outpatients; (2) ages of 16 to 60 years; (3) meeting the diagnostic criteria of DSM-IV^24^ with schizophrenia paranoid type, MDD, bipolar I disorder, manic episode, bipolar I disorder, depressed episode and PD, respectively. The exclusion criteria for patients: (1) in pregnancy or the puerperium; (2) younger than 16 years or older than 60 years; (3) with secondary mental disorders, such as mental disorders occurred on the basis of the use of certain drugs, with organic brain disease or severe physical illness; (4) current incidents of alcohol or drug abuse and dependence, or any other primary neurological illness, including dementia or stroke; (5) with serious cardiac, pulmonary, hepatic or renal diseases or any type of tumor. All patients except for 8 PD and 7 BD had been drug-free for at least 2 weeks before entering the trial. The HCs without any history of DSM-IV Axis I disorders, positive family history of mental disorders and severe physical disorders were recruited from Affiliated ZhongDa Hospital of Southeast University Medical Examination Center during the same time frame for this study.

The baseline clinical data of patients were collected in detail, including age, gender, marital status, education, frequency and duration of illness, occupation, body mass index (BMI), cigarette smoking, alcohol consumption, family history, comorbidities (for example, hyperlipidaemia, hypertension, diabetes mellitus, *et al*.) and the scores of multiple scales. On admission, two trained and experienced senior psychiatrists conducted the assessments of different scales. Scale for Assessment Positive Symptom (SAPS)^25^ was used to assess the positive symptom of SZ patients, 17-item Hamilton Depression Rating Scale (HDRS)^26^ was used to assess the depressive symptom of patients with MDD and BD, Young Mania Rating Scale (YMRS)^27^ was used to estimate the manic symptoms of BM patients and Hamilton Anxiety Rating Scale (HARS)^28^ was used to estimate anxious symptoms of PD patients by psychiatrists, respectively. While Panic Disorder Severity Scale (PDSS)^29^ was applied to PD patients for assessing the severity of their symptoms by themselves. SAPS, HDRS, YMRS, HARS and PDSS were all used to estimate whether HCs have any mental symptom, and all of these scales with HCs were normal. Age, gender, BMI and other important variables of participants from above different hospitals were statistically analyzed, and the results were not significant differences.

Written informed consents were provided by the participants or their legal guardians after they were given a description of the study in accordance with the Declaration of Helsinki. All procedures of this study were approved by the Ethics Committee of the Affiliated ZhongDa Hospital of Southeast University.

### Sample collection

Venous blood samples (5 mL) were collected in coagulant tubes with gel from fasting subjects between 06:30 and 08:00. The blood samples were left to facilitate clotting at room temperature, then centrifuged at 3500 rpm for 10 minutes to obtain serum. After that, aliquots (0.5 mL) were taken and stored at −86 °C until biochemical analyses.

### Protein Assays

Double antibody sandwich enzyme-linked immunosorbent assay (ELISA) was used to measure serum protein concentrations of tPA, PAI-1, BDNF, proBDNF, TrkB and p75NTR. The human tPA, PAI-1 and BDNF levels were measured by ELISA kits [tPA (DTPA00; R&D Systems; USA); PAI-1 (DSE100; R&D Systems; USA); BDNF (DBD00, R&D Systems; USA)] and proBDNF, TrkB and p75NTR levels were assessed using DuoSet human ELISA Development System (proBDNF: DY3175, TrkB: DYC397-2, p75NTR: DY367 and Ancillary Reagent: DY008; R&D Systems; USA). Protocols were performed according to the manufacturer’s instructions. In order to minimize assay variance, each protein concentrations of all subjects were measured on the same day. All experiments were performed in duplicate. The sensitivity of tPA, PAI-1 and BDNF assay were 1.40-16.1 pg/mL, 0.014-0.142 ng/ml and less than 20 pg/mL, respectively; while the sensitivity of proBDNF, TrkB and p75NTR assay were not given exactly in the specification. No significant cross-reactivity or interference was observed in each assay.

### Statistical analysis

All statistical computations were performed with SPSS (version 22, IBM, Armonk, NY, USA). The data were expressed as the mean ± standard deviation (M ± SD). D’Agostino and Pearson omnibus normality test was used to the exam for normality of data. Discrete variables, such as gender were performed with Chi-square (*χ* ^*2*^) test. One-way analysis of variance (ANOVA) (for normal distributions) or nonparametric test (Kruskal-Wallis H test, for non-normal distribution) was used to compare with continuous variables among different study groups as applicable. Kruskal-Wallis ANOVA test was used to compare differences in various protein levels among different study groups, and a Dunn-Bonferroni test for post hoc comparisons. The abnormal data determined by M ± 3 SD were excluded. To differentiate SZ, MDD, BM, BD, PD patients and HCs, we evaluated the discriminatory capacity of the combinations of these six proteins in every two groups by calculating the area under the receiver operating characteristic (ROC) curve (AUC) (AUC: 0.9–1 = excellent; 0.8–0.9 = good; 0.7–0.8 = fair; 0.6–0.7 = poor; 0.5–0.6 = fail)^21^ using logistic regression, sensitivity and specificity. Statistical significance was set at a 2-tailed *P* < 0.05 for all statistical tests.

## Results

### Study population

The demographic and clinical characteristics of all participants are showed in Table 1. No significant differences were observed among the six groups for gender, age, education levels, BMI and marriage status. However, there were significant differences in duration of illness (F _(4, 141)_ = 9.513, *P* < 0.001) and family history (*χ* ^*2*^_Kruskal-Wallis, df(4)_ = 17.579, *P* = 0.001) among five patient groups.

**Table 1:** Demographic and clinical characteristics of participants.

Variable	SZ	MDD	BM	BD	PD	HCs	F/*χ* ^*2*^	*P*-value
Age^a^, years (range)	34.35 ± 10.89 (17–60)	41.60 ± 12.43 (18–57)	34.40 ± 11.63 (20–60)	34.18 ± 13.67 (18–60)	35.50 ± 12.38 (16–56)	35.60 ± 12.90 (20–57)	1.593	0.165
Gender^b^, male/female, n (total N)	15/19 (N = 34)	5/25 (N = 30)	14/16 (N = 30)	11/11 (N = 22)	15/15 (N = 30)	16/14 (N = 30)	10.940	0.053
BMI^a^, kg/m^2^	22.32 ± 2.85	22.06 ± 2.59	22.71 ± 3.42	24.10 ± 4.05	22.48 ± 2.76	22.89 ± 1.02	1.529	0.183
Education^a^, years	10.62 ± 3.31	12.07 ± 3.50	12.07 ± 3.02	10.73 ± 4.21	12.77 ± 2.92	11.67 ± 3.66	1.755	0.125
Marital status^b^							3.555	0.615
Single, n	15	5	9	8	7	10		
Married, n	15	24	19	12	20	18		
Divorce/widow, n	4	1	2	2	3	2		
Family history of psychosis^b^, yes/no	14/20	3/27	6/24	9/13	2/28	0/30	28.782	< 0.001**
Duration of illness^a^, years	6.06 ± 6.16	2.78 ± 2.95	10.44 ± 9.91	9.25 ± 8.72	1.73 ± 3.11	—	9.513	< 0.001**
Drug-free, n	34	30	30	15	22	30		
Drug	—	—	—	7	8	—		
Antidepressants, n	—	—	—	5	6	—		
Valproate/Lithium, n	—	—	-	7	—	—		
Antipsychotics, n	—	—	—	4	—	—		
Benzodiazepines, n	—	—	—	3	8	—		
SAPS total score	42.59 ± 11.06	—	—	—	—	0.37 ± 0.67		
SAPS comprehensive evaluation score	12.29 ± 2.71	—	—	—	—	0.27 ± 0.45		
HARS score	—	—	—	—	17.37 ± 6.12	0.83 ± 0.87		
HDRS score	—	24.07 ± 4.32	—	22.00 ± 4.54	—	1.40 ± 0.82		
YMRS score	—	—	32.00 ± 10.43	—	—	0.67 ± 0.71		
PDSS score	—	—	—	—	12.70 ± 4.53	0.70 ± 0.70		

Note: Abbreviations: SZ, schizophrenia; MDD, major depressive disorder; BM, bipolar mania; BD, bipolar depression; PD, panic disorder; HCs, healthy controls; BMI, body mass index; SAPS, Scale for Assessment Positive Symptom; HARS, Hamilton Anxiety Rating Scale; HDRS, 17-item Hamilton Depression Rating Scale; YMRS, Young Mania Rating Scale; PDSS, Panic Disorder Severity Scale. Symbol: -, no data. Comparison among six groups: ^a^one-way ANOVA analysis, ^b^Chi-square test.

### Serum proteins levels in different groups of patients

**Figure 1:**
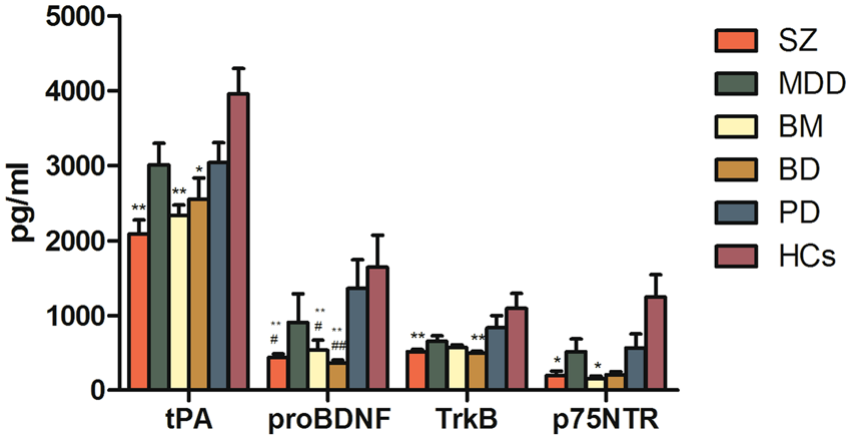
Serum concentrations of four proteins in patients with different disorders and HCs. Abbreviations: SZ, schizophrenia paranoid type; MDD, major depressive disorder; BM, bipolar mania; BD, bipolar depression; PD, panic disorder; HCs, healthy controls; tPA, tissue plasminogen activator; proBDNF, precursor brain-derived neurotrophic factor; TrkB, tropomyosin-related kinase B; p75NTR, neurotrophin receptor p75. Compared with HCs group by Kruskal-Wallis test followed by Dunnett T3 test, *P < 0.05, **P < 0.01. Compared with PD group by Kruskal-Wallis test followed by Dunnett T3 test, ^#^ *P* < 0.05, ^##^ *P* < 0.01. Several extreme values which below the minimum detectable concentration of the kit or higher than three standard deviations were removed out from tPA, proBDNF, TrkB and p75NTR. After removing out the extreme values, the remaining number of cases showed as follows: tPA (33 SZ, 30 MDD, 29 BM, 22 BD, 30 PD and 30HCs), proBDNF (32 SZ, 28 MDD, 29 BM, 21 BD, 28 PD and 28 HCs), TrkB (33 SZ, 29 MDD, 29 BM, 21 BD, 29 PD and 29 HCs), p75NTR (27 SZ, 26 MDD, 23 BM, 19 BD, 25 PD and 27 HCs). Finally, 27 SZ, 25 MDD, 22 BM, 19 BD, 24 PD patients and 25 HCs had all the four proteins.

**Figure 2:**
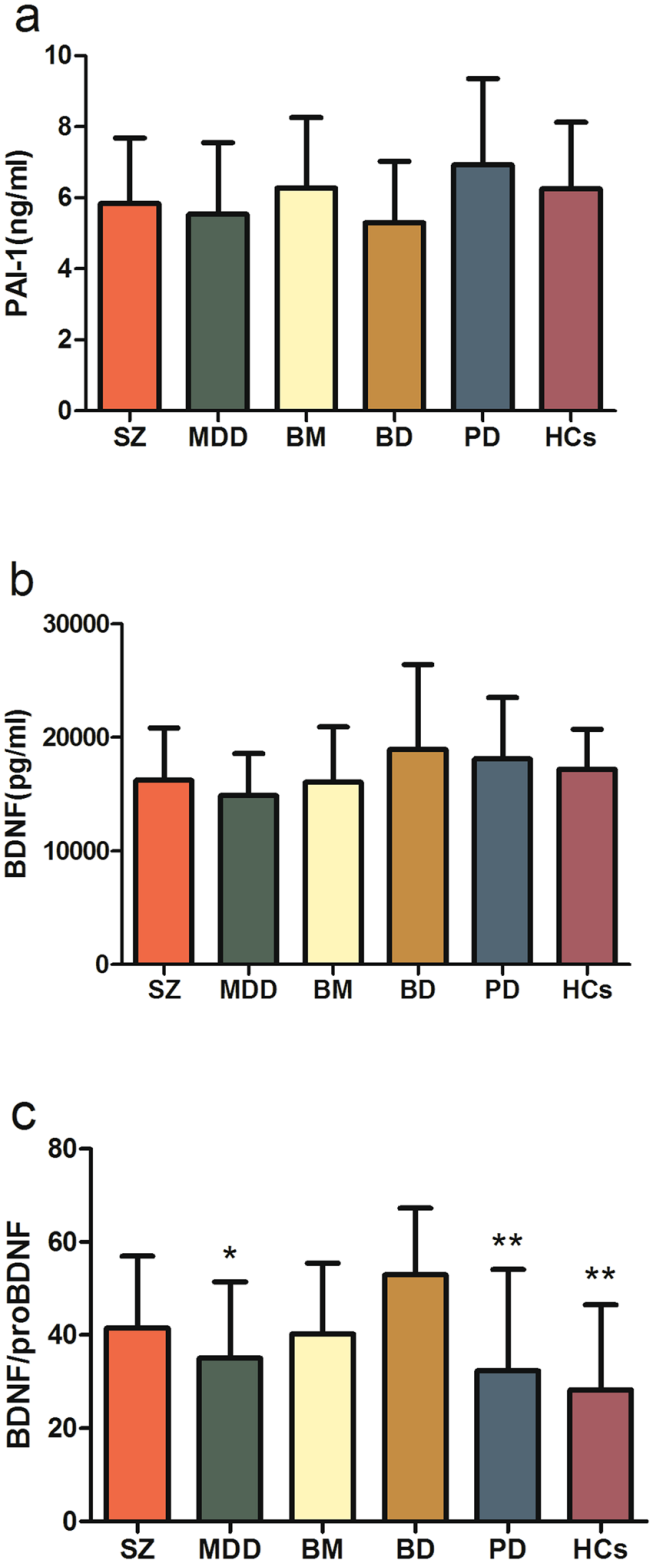
Serum concentrations of PAI-1 and BDNF and BDNF/proBDNF ratio in patients with different disorders and HCs. (**a**) PAI-1serum concentrations in patients with different disorders and HCs. (**b**) BDNF serum concentrations in patients with different disorders and HCs. (**c**) The value of BDNF/proBDNF in patients with different disorders and HCs. Abbreviations: SZ, schizophrenia; MDD, major depressive disorder; BM, bipolar mania; BD, bipolar depression; PD, panic disorder; HCs, healthy controls; PAI-1, plasminogen activator inhibitor-1; BDNF, brain-derived neurotrophic factor. Compared with BD group by Kruskal-Wallis test followed by Bonferroni test, *P < 0.05, **P < 0.01. Several extreme values which higher than three standard deviations were removed out from PAI-1 and the value of BDNF/proBDNF. After removing out the extreme values, the remaining number of cases showed as follows: PAI-1 (34 SZ, 30 MDD, 30 BM, 22 BD, 29 PD and 30 HCs) and BDNF/proBDNF (32 SZ, 28 MDD, 29 BM, 21 BD, 28 PD and 28 HCs).

The levels of the six serum proteins (tPA, PAI-1, BDNF, proBDNF, TrkB and p75NTR) and the value of BDNF/proBDNF in SZ, MDD, BM, BD and PD patients and HCs were shown in Figs 1 and 2. Serum levels of p75NTR were below the minimum detectable concentration of the kit in 24 subjects, and higher than three SD in five participants. All of these values were removed together with the extreme values of other proteins. 27 SZ, 25 MDD, 22 BM, 19 BD, 24 PD patients and 25 HCs had all six proteins and BDNF/proBDNF ratio. Significant differences for four serum protein concentrations and the value of BDNF/proBDNF among these groups were revealed by Kruskal-Wallis H nonparametric test (tPA, *χ* ^*2*^_Kruskal-Wallis, df(5)_ = 27.202, *P* = 0.001; proBDNF, *χ* ^*2*^_Kruskal-Wallis, df(5)_ = 36.667, *P* < 0.001; TrkB, *χ* ^*2*^_Kruskal-Wallis, df(5)_ = 20.946, *P* = 0.001; p75NTR, *χ* ^*2*^_Kruskal-Wallis, df(5)_ = 15.433, *P* = 0.009; BDNF/proBDNF, *χ* ^*2*^_Kruskal-Wallis, df(5)_ = 24.384, *P* < 0.001). However, serum PAI-1 and BDNF levels were not significantly different among the six groups (*χ* ^*2*^_Kruskal-Wallis, df(5)_ = 10.365, *P*_ = _0.066; *χ* ^*2*^_Kruskal-Wallis, df(5)_ = 10.812, *P* = 0.055, respectively). Nonparametric multiple comparison tests further showed that, compared with HCs, serum levels of tPA and proBDNF were significantly lower in SZ, BM and BD groups (Kruskal-Wallis test followed by Bonferroni test, all *P* < 0.05), but not in MDD and PD groups. TrkB levels were significantly lower in SZ and BD groups (Kruskal-Wallis test followed by Dunnett T3 test, *P* < 0.01 for both), while p75NTR levels were declined in patients with SZ and BM (Kruskal-Wallis test followed by Dunnett T3 test, both *P* < 0.05) compared with HCs. Besides, serum proBDNF levels in SZ, BM and BD groups were also down-regulated with statistical significance compared to PD group (Kruskal-Wallis test followed by Dunnett T3 test, all *P* < 0.05). Furthermore, the ratio of BDNF to proBDNF in BD group was highest in all groups (Kruskal-Wallis test followed by Bonferroni test, all *P* < 0.05).

### Correlations of serum protein levels with clinical variables in patients with different mental disorders

The results of Pearson’s correlation analysis in SZ patients showed that there was positive correlation between tPA and comprehensive evaluation scores of SAPS (*r* = 0.352, *P* = 0.044) as well as PAI-1 and BMI (*r* = 0.358, *P* = 0.038), and negative correlation between BDNF and total scores of SAPS (*r* = −0.344, *P* = 0.047).

In MDD patients, tPA and BDNF/proBDNF ratio was negatively correlated with BMI and HDRS scores (*r* = −0.565, *P* = 0.001 and *r* = −0.403, *P* = 0.033, respectively). tPA had negative correlation with the episode of BM (*r* = −0.423, *P* = 0.022) and BDNF/proBDNF ratio had a negative correlation with age (*r* = −0.388, *P* = 0.037), while p75NTR was positively correlated with the duration of illness in BM patients (*r* = 0.503, *P* = 0.014).

In patients of BD, tPA and p75NTR were positively correlated with the duration of illness and BMI (*r* = 0.356, *P* = 0.023 and *r* = 0.429, *P* = 0.011, respectively). TrkB had a negative correlation with age (*r* = −0.443, *P* = 0.006), and a positive correlation with BMI and education level (*r = *0.387, *P* = 0.015; *r = *0.330, *P* = 0.048, respectively).

There was a positive correlation between TrkB and PDSS scores (*r = *0.393, *P* = 0.035), and between BDNF/proBDNF value and age (*r = *0.406, *P* = 0.032), and a negative association between p75NTR and BMI (*r* = −0.546, *P* = 0.005) and the value of BDNF/proBDNF and education (*r* = −0.391, *P* = 0.040) in PD patients.

No other correlation was found among these serum protein concentrations, BDNF/proBDNF ratio and clinical characteristics in all groups (Supplementary Table 1).

### A single and combined protein level in differentiation of mental disorders

**Figure 3:**
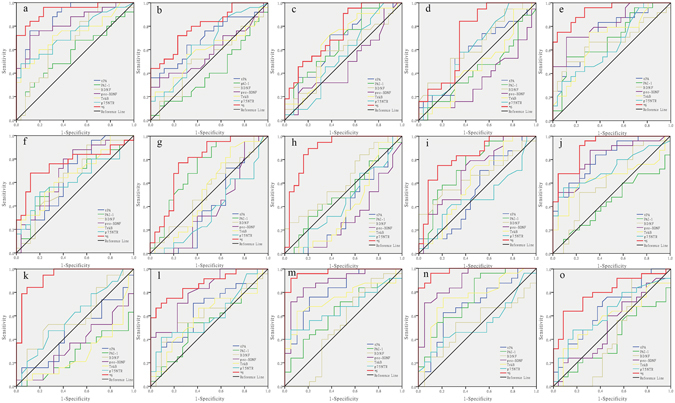
Diagnostic and differential powers of these six serum proteins. ROC curves of each protein and their combination between: (**a**) SZ and HCs. (**b**) SZ and MDD. (**c**) SZ and BM. (**d**) SZ and BD. (**e**) SZ and PD. (**f**) MDD and HCs. (**g**) MDD and BM. (**h**) MDD and BD. (**i**) MDD and PD. (**j**) BM and HCs. (**k**) BM and BD. (**l**) BM and PD. (**m**) BD and HCs. (**n**) BD and PD. (**o**) PD and HCs. ROC, the receiver operative characteristic.

We analyzed the diagnostic and differential power of single tPA, PAI-1, BDNF, proBDNF, TrkB and p75NTR level in the tPA-BDNF pathway, and the combination of six proteins levels by the ROC analysis. Figure 3a–o presents the ROC curves for the serum levels of these proteins alone and their combination between different groups.

As we can see, single tPA, proBDNF, TrkB and p75NTR could help differentiate SZ from HCs (AUC = 0.886, 0.828, 0.735 and 0.739, respectively). tPA and proBDNF also yielded fair to good differential efficacy between SZ and PD (AUC = 0.747 and 0.821, respectively), but single protein failed to differentiate SZ from MDD, BM and BD. However, the combination of tPA, PAI-1, BDNF, proBDNF, TrkB and p75NTR yield an excellent diagnostic value between SZ and HCs (AUC = 0.935, sensitivity = 0.960 and specificity = 0.769) and SZ and PD (AUC = 0.933, sensitivity = 0.750 and specificity = 0.962), and a fair differential efficacy between SZ and MDD (AUC = 0.798, sensitivity = 0.720 and specificity = 0.769), and SZ and BM (AUC = 0.745, sensitivity = 0.955 and specificity = 0.462), and SZ and BD (AUC = 0.721, sensitivity = 0.842 and specificity = 0.654).

No single protein could differentiate MDD from HCs, all of them had a poor diagnostic power with AUC from 0.623 to 0.699. However, combining tPA, PAI-1, BDNF, proBDNF, TrkB and p75NTR could differentiate MDD from HCs and other mental illness. The combination yielded fair to good efficacy for differentiating MDD patients from HCs (AUC = 0.763, sensitivity = 0.680 and specificity = 0.880), BM (AUC = 0.780, sensitivity = 0.773 and specificity = 0.760), BD (AUC = 0.893, sensitivity = 0.895 and specificity = 0.800) and PD (AUC = 0.825, sensitivity = 0.750 and specificity = 0.840).

tPA and proBDNF yielded good diagnostic power for differentiating BM and BD form HCs (tPA: AUC = 0.813 for BM and AUC = 0.815 for BD; proBDNF: AUC = 0.813 for BM and AUC = 0.886 for BD); while p75NTR and TrkB provided a fair differential efficacy with AUC = 0.719 and 0.753 for BM and BD, respectively. ProBDNF also yielded a good differential efficacy between BM and PD (AUC = 0.808), and BD and PD (AUC = 0.894), PAI-1 and TrkB could distinguish BD and PD (AUC = 0.750 and 0.755, respectively), but not BM and PD. However, none of these proteins alone could differentiate BM and BD (all AUC < 0.6). The ROC analysis indicated that the combination yielded an excellent diagnostic efficacy for differentiating BM and BD from HCs (AUC = 0.909, sensitivity = 0.920 and specificity = 0.773, and AUC = 0.975, sensitivity = 0.920 and specificity = 0.947, respectively) as well as BM from BD (AUC = 0.935, sensitivity = 0.789 and specificity = 0.955). Moreover, the combination could also differentiate BM and BD from PD patients (AUC = 0.871, sensitivity = 0.667 and specificity = 0.955; AUC = 0.978, sensitivity = 0.958 and specificity = 0.895, respectively).

Although some of these proteins could distinguish other mental disorders from PD alone, none of them could differentiate PD from HCs (all AUC < 0.6). However, the combination could differentiate PD from HCs with the AUC = 0.802, sensitivity = 0.640 and specificity = 0.917 (Supplementary Table 2).

## Discussion

In present study, we measured serum levels of various proteins involved in tPA-BDNF pathway from patients with a variety of mental disorders, including SZ, MDD, BM, BD and HC, and tested if a combination of serum levels of multiple proteins could aid diagnosis and differentiation of mental disorders. ROC analysis indicated that the combination could be used to improve the diagnosis and differentiation.

Many studies investigated the serum or plasma level of single protein like BDNF in SZ or mood disorders, and suggested a potential role of BDNF as a biomarker of disease activity and treatment response^30–40^. However, only a few studies were performed to test the role of a combination of multiple proteins in diagnosis and differentiation of various mental disorders. In our previous study, we reported that the disturbance of serum tPA-BDNF pathway was related to the pathogenesis of MDD, and the combination of tPA, BDNF, proBDNF, TrkB and p75NTR, the factors found in the pathway, could improve diagnostic power from a single protein for MDD diagnosis^21^. In this research, we tested if the same strategy could apply to other mental disorders. Indeed, consistent with previous work, we found that the combination of six proteins was better in diagnosis and differentiation of mental disorders than any of single protein.

We found that there were no significant differences in serum BDNF levels in SZ, MDD, BM, BD and PD patients from HCs. The results are in agreement with several previous studies^41–44^, but some previous works also showed a decrease in peripheral BDNF concentrations in SZ^37^, MDD^13, 21^ and bipolar disorder^38^. We speculated that the different samples may be the reasons for these conflicts of the results. These results indicate that single serum BDNF level may be not helpful for the diagnosis and differentiation of mental disorders.

While serum TrkB level was lower in patient groups than in HCs in our study, it reached a statistical significance only in SZ and BD groups. The change of TrkB in SZ patients in our study was consistent with one previous report showing that TrkB level was significantly lower in SZ patients^42^. Our results, however, showed no significant difference in TrkB level between MDD and BM, which is inconsistent with previous reports^15, 45^. We think the discrepancies could be due to or related to the different samples. Also, we found that TrkB level was statistically lower in BD patients, while not in PD compared with HCs.

We found that proBDNF levels were down regulated in SZ, BM and BD, but not in MDD and PD, while p75NTR levels were reduced in SZ and BM patients. The results were consistent with previous reports that proBDNF levels were significantly lower in bipolar disorder patients^46^ and tended to be low in chronic SZ patients^47^, but not in agreement in MDD patients^21^. In previous study, we found serum levels of proBDNF and p75NTR were higher in MDD patients, which was in agreement with another previous research^16^. The exact reason of this discrepancy is unknown, we speculate that the different ratio of male to female in MDD subjects may be one reason.

The value of BDNF/proBDNF in the present study was higher in BD patients than MDD, PD and HCs. Although the results were not always consistent with previous studies due to the different samples and methodology, they still indicated that the BDNF/proBDNF ratio or the conversion of proBDNF to mature BDNF might be abnormal in mental disorders that resulted in similar decline of serum proBDNF and p75NTR.

Our results that tPA serum level decreased significantly in SZ patients compared with HCs and PAI-1 level between SZ patients and HCs was not significantly different, which was consistent with previous studies^48, 49^, suggested that tPA was associated with the pathogenesis of SZ. We also found that serum levels of tPA in BM and BD patients were significantly lower than HCs, indicating that tPA may be also involved in the pathogenesis of bipolar disorder. However, we did not found there was any significant difference in PAI-1 levels among the six study groups. This may be explained by the study which concluded that serum levels of PAI-1 were independent of MDD or SZ but related to the metabolic syndrome^50, 51^.

In addition to the studies that investigated the diagnostic value of peripheral protein levels, some studies also investigated the correlations of serum protein levels with clinical variables in patients with different mental disorder. Karegea *et al*.^52^ found that serum BDNF levels were negatively correlated with the Montgomery Asberg Depression Rating Scale scores. Zhou *et al*.^16^ found that BDNF and TrkB were negatively correlated with HRSD scores, while proBDNF and p75NTR had positive correlations with the scores of HRSD. Zhao and colleagues^53^ did not find any association between protein levels and the severity of symptoms, but they observed plasma proBDNF levels had negative correlations with age and age of the first onset, and BDNF/proBDNF ratio was positively correlated with age and age of the first onset in MDD patients. In present study, we found that the severity of depressive symptoms was not correlated with BDNF and TrkB, nor proBDNF and p75NTR. But it was negatively correlated with BDNF/proBDNF ratio. This indicated that the abnormality of BDNF and proBDNF levels might not mediate pathogenesis of MDD, but the imbalance of the BDNF/proBDNF might be involved in.

We also found that tPA had a positive correlation with the comprehensive evaluation scores of SAPS, and BDNF had a negative correlation with the total scores of SAPS, which indicated that the tPA-BDNF pathway may play an important role in the pathophysiological mechanism of SZ.

Lasić *et al*.^50^ reported that in patients with SZ and MDD, PAI-1 was not correlated with their psychiatric diagnosis, but with the metabolic syndrome. In our research, tPA was negatively correlated with BMI of MDD patients, and PAI-1 was positively correlated with BMI of SZ patients, which were consistent with the previous report^50^.

Our previous research suggested that the serum tPA-BDNF pathway was involved in MDD pathogenesis and each protein in the pathway might be a potential biomarker for MDD diagnosis^21^. The findings in the present study indicated that each serum level of tPA, PAI-1, BDNF, proBDNF, TrkB and p75NTR in tPA-BDNF pathway might yield a relatively high level of diagnostic power, but they failed to play a role in differentiation of mental disorders. This result may be explained by that both MDD and BD showed symptoms of depression, or by the similar symptoms among different mental disorders. However, accurate differentiation of psychiatric disorders is essential for treatment and prognosis of patients. Previous studies found that the combinations of peripheral protein levels could differentiate MDD and bipolar disorder, but not MDD and BD^18, 19^. Our present results implied that the combination of serum tPA, PAI-1, BDNF, proBDNF, TrkB and p75NTR in tPA-BDNF pathway yield high differential power between MDD and BD (AUC = 0.893, sensitivity = 0.895 and specificity = 0.800). Furthermore, this combination also help differentiation between any two groups, particularly for BD and PD, with AUC as high as 0.978, 95.8% of sensitivity and 89.5% of specificity. Thus, the combination of multiple serum protein may aid differentiating psychiatric disorders. The total measurements of serum tPA, PAI-1, BDNF, proBDNF, TrkB and p75NTR concentrations were suggested to be a promising assistance for diagnosing mental illness accurately in the early stage. As far as we know, this is the first diagnostic and differential panel that has a potential to be used in clinical differentiation and diagnosis of mental disorders.

Limitations in this study should be considered. First, the study contained a small sample size for each group and did not include the data of post-treatment. Second, although most of patients were drug-free for at least two weeks, there were eight PD and seven BD patients who had been taken drugs before they entered into the trial, the medication may affect serum protein concentrations and we did not know whether the medicine was still effective and the degree of their effects. Third, there were more females than males in MDD group and their ages were older than other subgroups, which may impact the mean levels of the six proteins. Fourth, the selected research subjects come from different hospitals, which may lead to a lack of homogeneity on the severity of illness. In future studies, we should measure the serum tPA-BDNF pathway protein levels in large drug-naïve and well-matched samples and add a post-treatment study, we should further test the differential power of this combination in patulous types of illness, including the anxiety spectrum disorders, different types of schizophrenia and other psychiatric disorders.

## Electronic supplementary material

Supplementary Table 1

Supplementary Table 2

Supplementary Figure
