# Supplementary material for: Combined serum levels of multiple proteins in tPA-BDNF pathway may aid the diagnosis of five mental disorders
Source: Sci Rep. 2017 Jul 31;7:6871. doi: 10.1038/s41598-017-06832-6 (PMC5537244; doi:10.1038/s41598-017-06832-6)
Supplement: Supplementary file 1 — Supplementary Table 1 [file 41598_2017_6832_MOESM1_ESM.pdf]

Combined serum levels of multiple proteins in tPA-BDNF pathway may aid the diagnosis of five mental disorders

Suzhen Chen, MD[1,2], Haitang Jiang, MD[1,2], Yang Liu, BD[3], Zhenhua Hou, MD[1,2], Yingying Yue, MD[1,2], Yuqun Zhang, MD[1,2], Fuying Zhao, MD[1,2], Zhi Xu, PhD[1,2], Yinghui Li, MD[1,2], Xiaodong Mou, PhD[1,2], Lei Li, MD[1,2], Tianyu Wang, MD[1,2], Jingjing Zhao, BD[4], Chongyang Han, MD[4], Yuxiu Sui, PhD[4], Ming Wang, BD[5], Zhong Yang, BD[5], Yan Lu, BD[6], Yifeng Zhu, BD[6], Jianhua Li, MD[7], Xinhua Shen, MD[7], Fei Sun, BD[8], Qingsong Chen, BD[8], Huanxin Chen, PhD[9] & Yonggui Yuan, PhD[1,2*]

[1]Department of Psychosomatics and Psychiatry, ZhongDa Hospital, Medical School of Southeast University, Nanjing 210009, PR China.

[2]Institute of Psychosomatics, Medical School of Southeast University, Nanjing 210009, PR China.

[3]Institute of Neuropsychiatric, Brain Hospital, Nanjing Medical University, Nanjing 210029, PR China.

[4]Department of Psychiatry, Brain Hospital, Nanjing Medical University, Nanjing 210029, PR China.

[5] Department of Psychiatry, The Third People's Hospital of Changshu, Suzhou 215500, PR China.

[6]Department of Psychiatry, The Fourth People's Hospital of Zhangjiagang, Suzhou 215600, PR China.

[7]Department of Psychiatry, The Third People's Hospital of Huzhou, Huzhou 313000, PR China.

[8]Department of Psychiatry, The Second People's Hospital of Jingjiang, Taizhou 214500, PR China.

[9]Key Laboratory of Cognition and Personality, Ministry of Education; School of Psychology, Southwest University, Chongqing 400175, PR China.

*Corresponding author at: Department of Psychosomatics and Psychiatry, ZhongDa Hospital, Medical School of Southeast University, Nanjing 210009, PR China.

Tel./fax:+86 25 83285124; E-mail address: yygylh2000@sina.com(Y. Yuan).

Supplementary Table 1. Correlation analysis among tPA, PAI-1, BDNF, proBDNF, TrkB, p75NTR, BDNF/proBDNF ratio and clinical variables in different disorders.

Disorders	Clinical variables	tPA	PAI-1	BDNF	proBDNF	TrkB	p75NTR	BDNF/proBDNF ratio
SZ	SAPS total score	*r*=0.215, *P*=0.231	*r*=-0.108, *P*=0.542	***r*=-0.344, *P*=0.047***	*r*=0.035, *P*=0.851	*r*=-0.236, *P*=0.186	*r*=-0.020, *P*=0.920	*r*=-0.232, *P*=0.202
	SAPS comprehensive evaluation score	***r*=0.352, *P*=0.044***	*r*=-0.124, *P*=0.484	*r*=-0.248, *P*=0.157	*r*=-0.022, *P*=0.903	*r*=-0.305, *P*=0.084	*r*=0.220, *P*=0.584	*r*=-0.183, *P*=0.316
	Age	*r*=0.233, *P*=0.191	*r*=-0.155, *P*=0.381	*r*=0.079, *P*=0.659	*r*=0.003, *P*=0.989	*r*=0.224, *P*=0.210	*r*=0.208, *P*=0.298	*r*=-0.095, *P*=0.605
	BMI	*r*=0.124, *P*=0.492	***r*=0.358, *P*=0.038***	*r*=-0.187, *P*=0.290	*r*=-0.161, *P*=0.378	*r*=-0.224, *P*=0.209	*r*=-0.277, *P*=0.161	*r*=-0.104, *P*=0.571
	Education	*r*=-0.007, *P*=0.970	*r*=0.098, *P*=0.581	*r*=0.188, *P*=0.286	*r*=-0.149, *P*=0.416	*r*=0.119, *P*=0.509	*r*=0.283, *P*=0.153	*r*=0.187, *P*=0.306
	Duration of illness	*r*=-0.075, *P*=0.676	*r*=0.022, *P*=0.902	*r*=-0.129, *P*=0.468	*r*=-0.121, *P*=0.510	*r*=0.147, *P*=0.414	*r*=-0.141, *P*=0.483	*r*=0.039, *P*=0.832
MDD	HDRS score	*r*=-0.125, *P*=0.511	*r*=0.038, *P*=0.840	*r*=-0.165, *P*=0.383	*r*=0.352, *P*=0.067	*r*=0.181, *P*=0.349	*r*=0.233, *P*=0.252	***r*=-.0403, *P*=0.033***
	Age	*r*=0.130, *P*=0.494	*r*=-0.195, *P*=0.301	*r*=-0.030, *P*=0.876	*r*=0.115, *P*=0.559	*r*=-0231, *P*=0.229	*r*=-0.171, *P*=0.403	*r*=-0.020, *P*=0.920
	BMI	***r*=-0.565, *P*=0.001***	*r*=-0.066, *P*=0.728	*r*=0.159, *P*=0.403	*r*=0.096, *P*=0.629	*r*=0.004, *P*=0.984	*r*=-0.377, *P*=0.057	*r*=-0.130, *P*=0.511
	Education	*r*=-0.190, *P*=0.315	*r*=0.206, *P*=0.274	*r*=-0.240, *P*=0.202	*r*=0.245, *P*=0.209	*r*=0.266, *P*=0.162	*r*=0.232, *P*=0.255	*r*=-0.205, *P*=0.296
	Duration of illness	*r*=0.153, *P*=0.420	*r*=-0.087, *P*=0.648	*r*=-0.115, *P*=0.546	*r*=-0.225, *P*=0.249	*r*=-0.233, *P*=0.224	*r*=-0.263, *P*=0.194	*r*=0.268, *P*=0.168
	Episodes	*r*=0.106, *P*=0.577	*r*=-0.077, *P*=0.685	*r*=0.106, *P*=0.577	*r*=0.105, *P*=0.581	*r*=0.017, *P*=0.933	*r*=0.026, *P*=0.893	*r*=-0.121, *P*=0.555
BM	YMRS score	*r*=-0.156, *P*=0.418	*r*=-0.239, *P*=0.204	*r*=0.222, *P*=0.239	*r*=0.199, *P*=0.301	*r*=0.187, *P*=0.330	*r*=0.071, *P*=0.748	*r*=-0.031, *P*=0.872

	Age	r=-0.095, P=0.624	r=-0.284, P=0.128	r=-0.281, P=0.132	r=-0.067, P=0.729	r=-0.313, P=0.098	r=-0.273, P=0.207	**r=-0.388, P=0.037***
	BMI	r=-0.103, P=0.595	r=0.084, P=0.660	r=0.127, P=0.502	r=0.144, P=0.456	r=-0.201, P=0.297	r=-0.152, P=0.490	r=-0.096, P=0.619
	Education	r=-0.036, P=0.853	r=-0.046, P=0.809	r=0.070, P=0.714	r=0.147, P=0.446	r=0.194, P=0.314	r=0.043, P=0.844	r=0.114, P=0.557
	Duration of illness	r=0.264, P=0.166	r=0.039, P=0.836	r=-0.093, P=0.625	r=-0.125, P=0.517	r=0.254, P=0.184	**r=0.503, P=0.014***	r=0.006, P=0.973
	Episodes	**r=-0.423, P=0.022***	r=-0.099, P=0.601	r=0.004, P=0.983	r=-0.308, P=0.105	r=-0.010, P=0.957	r=-0.407, P=0.054	r=0.156, P=0.419
BD	HDRS score	r=-0.098, P=0.532	r=0.219, P=0.164	r=0.156, P=0.320	r=0.158, P=0.329	r=-0.010, P=0.951	r=0.177, P=0.305	r=-0.069, P=0.670
	Age	r=0.044, P=0.778	r=0.013, P=0.932	r=-0.231, P=0.134	r=-0.063, P=0.694	**r=-0.443, P=0.006***	r=-0.089, P=0.599	r=-0.168, P=0.290
	BMI	r=0.156, P=0.310	r=0.247, P=0.108	r=0.177, P=0.248	r=0.115, P=0.468	**r=0.387, P=0.015***	**r=0.429, P=0.011***	r=-0.048, P=0.763
	Education	r=-0.028, P=0.863	r=0.042, P=0.796	r=0.023, P=0.886	r=0.194, P=0.241	**r=0.330, P=0.048***	r=0.138, P=0.432	r=-0.122, P=0.460
	Duration of illness	**r=0.356, P=0.023***	r=0.076, P=0.630	r=0.031, P=0.843	r=0.054, P=0.738	r=-0.303, P=0.063	r=-0.085, P=0.622	r=-0.064, P=0.693
	Episodes	r=0.206, P=0.358	r=-.143, P=.526	r=.003, P=.989	r=.266, P=.244	r=-.288, P=.205	r=-.112, P=.649	r=-.074, P=.751
PD	PDSS score	r=0.279, P=0.136	r=-0.118, P=0.535	r=-0.039, P=0.837	r=-0.263, P=0.176	**r=0.393, P=0.035***	r=0.260, P=0.210	r=-0.001, P=0.998
	HARS crore	r=-0.080, P=0.675	r=0.156, P=0.409	r=0.026, P=0.892	r=0.091, P=0.645	r=-0.045, P=0.817	r=0.141, P=0.502	r=-0.020, P=0.920
	Age	r=0.062, P=0.746	r=-0.160, P=0.397	r=-0.039, P=0.836	r=-0.281, P=0.147	r=-0.296, P=0.119	r=-0.304, P=0.139	**r=0.406, P=0.032***
	BMI	r=0.076, P=0.690	r=0.313, P=0.092	r=-0.041, P=0.831	r=0.117, P=0.554	r=-0.345, P=0.067	**r=-0.546, P=0.005***	r=0.073, P=0.712
	Education	r=-0.267, P=0.154	r=0.328, P=0.076	r=-0.182, P=0.335	r=0.277, P=0.153	r=-0.047, P=0.807	r=0.094, P=0.656	**r=-0.391, P=0.040***
	Duration of illness	r=0.021, P=0.912	r=0.042, P=0.826	r=-0.319, P=0.086	r=-0.119, P=0.548	r=-0.102, P=0.598	r=-0.194, P=0.353	r=-0.086, P=0.663

Note: Abbreviations: SZ, schizophrenia; MDD, major depressive disorder; BM, bipolar mania; BD, bipolar depression; PD, panic disorder; BMI, body mass index; SAPS, Scale for Assessment Positive Symptom; HARS, Hamilton Anxiety Rating Scale; HDRS, 17-item Hamilton Depression Rating Scale; YMRS, Young Mania Rating Scale; PDSS, Panic Disorder Severity Scale. tPA, tissue plasminogen activator; PAI-1, plasminogen activator inhibitor-1; BDNF, brain-derived neurotrophic factor; pro-BDNF, precursor-BDNF; TrkB, tropomyosin-related kinase B; p75NTR, neurotrophin receptor p75.
