# Supplementary material for: Combined serum levels of multiple proteins in tPA-BDNF pathway may aid the diagnosis of five mental disorders
Source: Sci Rep. 2017 Jul 31;7:6871. doi: 10.1038/s41598-017-06832-6 (PMC5537244; doi:10.1038/s41598-017-06832-6)
Supplement: Supplementary file 2 — Supplementary Table 2 [file 41598_2017_6832_MOESM2_ESM.pdf]

Combined serum levels of multiple proteins in tPA-BDNF pathway may aid the diagnosis of five mental disorders

Suzhen Chen, MD[1,2], Haitang Jiang, MD[1,2], Yang Liu, BD[3], Zhenhua Hou, MD[1,2], Yingying Yue, MD[1,2], Yuqun Zhang, MD[1,2], Fuying Zhao, MD[1,2], Zhi Xu, PhD[1,2], Yinghui Li, MD[1,2], Xiaodong Mou, PhD[1,2], Lei Li, MD[1,2], Tianyu Wang, MD[1,2], Jingjing Zhao, BD[4], Chongyang Han, MD[4], Yuxiu Sui, PhD[4], Ming Wang, BD[5], Zhong Yang, BD[5], Yan Lu, BD[6], Yifeng Zhu, BD[6], Jianhua Li, MD[7], Xinhua Shen, MD[7], Fei Sun, BD[8], Qingsong Chen, BD[8], Huanxin Chen, PhD[9] & Yonggui Yuan, PhD[1,2*]

[1]Department of Psychosomatics and Psychiatry, ZhongDa Hospital, Medical School of Southeast University, Nanjing 210009, PR China.

[2]Institute of Psychosomatics, Medical School of Southeast University, Nanjing 210009, PR China.

[3]Institute of Neuropsychiatric, Brain Hospital, Nanjing Medical University, Nanjing 210029, PR China.

[4]Department of Psychiatry, Brain Hospital, Nanjing Medical University, Nanjing 210029, PR China.

[5] Department of Psychiatry, The Third People's Hospital of Changshu, Suzhou 215500, PR China.

[6]Department of Psychiatry, The Fourth People's Hospital of Zhangjiagang, Suzhou 215600, PR China.

[7]Department of Psychiatry, The Third People's Hospital of Huzhou, Huzhou 313000, PR China.

[8]Department of Psychiatry, The Second People's Hospital of Jingjiang, Taizhou 214500, PR China.

[9]Key Laboratory of Cognition and Personality, Ministry of Education; School of Psychology, Southwest University, Chongqing 400175, PR China.

*Corresponding author at: Department of Psychosomatics and Psychiatry, ZhongDa Hospital, Medical School of Southeast University, Nanjing 210009, PR China.

Tel./fax:+86 25 83285124; E-mail address: yygylh2000@sina.com(Y. Yuan).

Supplementary Table 2. Results of ROC curve analysis among the six groups.

Comparison	Analysis	tPA (pg/ml)	PAI-1 (ng/ml)	BDNF (pg/ml)	proBDNF (pg/ml)	TrkB (pg/ml)	p75NTR (pg/ml)	×6
SZ vs HCs	Sensitivity	0.760	0.680	0.680	0.720	0.720	0.480	0.960
	Specificity	0.885	0.500	0.692	0.885	0.692	0.962	0.769
	AUC	0.886	0.566	0.674	0.828	0.735	0.739	0.935
	Cutoff	2920.170	5.390	16137.240	468.250	504.000	582.005	0.257
	95% CI[a]	0.799-0.973	0.405-0.727	0.523-0.824	0.713-0.942	0.594-0.875	0.602-0.877	0.868-1.000
SZ vs MDD	Sensitivity	0.360	0.160	0.520	0.360	0.560	0.640	0.720
	Specificity	1.000	0.885	0654	0.923	0.692	0.654	0.769
	AUC	0.696	0.410	0.522	0.581	0.581	0.643	0.798
	Cutoff	3801.405	7.180	15730.260	575.930	501.545	134.250	0.481
	95% CI[a]	0.549-0.843	0.252-0.568	0.360-0.683	0.419-0.743	0.419-0.742	0.491-0.795	0.678-0.919
SZ vs BM	Sensitivity	0.727	0.955	0.591	0.273	0.682	1.000	0.955
	Specificity	0.615	0.346	0.692	0.846	0.654	0.154	0.462
	AUC	0.650	0.662	0.578	0.475	0.656	0.541	0.745
	Cutoff	1872.525	4.820	15840.405	451.240	496.635	10.070	0.300
	95% CI[a]	0.494-0.807	0.508-0.815	0.412-0.744	0.306-0.643	0.497-0.814	0.376-0.706	0.608-0.882

SZ vs BD	Sensitivity	0.526	0.158	0.526	0.053	0.947	0.579	0.842
	Specificity	0.692	0.885	0.731	1.000	0.192	0.654	0.654
	AUC	0.574	0.426	0.637	0.334	0.467	0.618	0.721
	Cutoff	2166.495	7.310	17249.295	860.300	408.290	134.250	0.393
	95% CI[a]	0.400-0.747	0.253-0.600	0.472-0.802	0.170-0.498	0.294-0.639	0.455-0.782	0.570-0.871
SZ vs PD	Sensitivity	0.833	0.542	0.500	0.708	0.750	0.333	0.750
	Specificity	0.615	0.885	0.885	0.885	0.692	0.923	0.962
	AUC	0.747	0.695	0.670	0.821	0.690	0.641	0.933
	Cutoff	1895.625	7.110	18633.720	458.530	513.815	421.530	0.689
	95% CI[a]	0.610-0.883	0.547-0.842	0.513-0.827	0.705-0.936	0.541-0.838	0.487-0.795	0.868-0.997
MDD vs HCs	Sensitivity	0.760	0.480	0.920	0.800	0.520	0.480	0.680
	Specificity	0.560	0.800	0.400	0.600	0.760	0.840	0.880
	AUC	0.666	0.635	0.642	0.699	0.664	0.623	0.763
	Cutoff	2862.285	6.185	13616.925	390.840	636.520	645.865	0.552
	95% CI[a]	0.515-0.818	0.478-0.792	0.486-0.798	0.552-0.846	0.514-0.814	0.464-0.782	0.624-0.903
MDD vs BM	Sensitivity	0.727	0.818	0.773	0.955	0.909	1.000	0.773
	Specificity	0.360	0.600	0.360	0.200	0.320	0.040	0.760

	AUC	0.400	0.732	0.533	0.395	0.531	0.366	0.780
	Cutoff	1815.930	5.085	13144.110	291.560	447.555	5.865	0.544
	95% CI[a]	0.235-0.565	0.588-0.876	0.365-0.700	0.230-0.559	0.362-0.700	0.207-0.526	0.645-0.915
MDD vs BD	Sensitivity	1.000	0.316	0.263	0.000	0.947	1.000	0.895
	Specificity	0.040	0.800	0.960	1.000	0.200	0.080	0.800
	AUC	0.377	0.512	0.621	0.298	0.393	0.440	0.893
	Cutoff	593.175	6.165	21088.095	11173.170	405.835	17.870	0.419
	95% CI[a]	0.211-0.542	0.336-0.687	0.452-0.790	0.144-0.452	0.224-0.561	0.269-0.611	0.795-0.990
MDD vs PD	Sensitivity	0.833	0.833	0.542	0.792	0.750	0.417	0.750
	Specificity	0.360	0.600	0.800	0.560	0.520	0.720	0.840
	AUC	0.532	0.747	0.658	0.698	0.607	0.485	0.825
	Cutoff	1839.030	5.125	18035.535	407.860	521.180	264.780	0.566
	95% CI[a]	0.366-0.698	0.608-0.885	0.501-0.816	0.551-0.845	0.446-0.767	0.318-0.652	0.707-0.943
BM vs HCs	Sensitivity	0.840	0.120	0.520	0.880	0.600	0.560	0.920
	Specificity	0.636	0.909	0.773	0.682	0.773	0.909	0.773
	AUC	0.813	0.434	0.609	0.813	0.643	0.719	0.909
	Cutoff	2357.310	9.115	17687.880	410.290	607.075	264.780	0.372

	95% CI[a]	0.693-0.932	0.268-0.599	0.443-0.775	0.689-0.936	0.479-0.806	0.571-0.867	0.827-0.991
BM vs BD	Sensitivity	0.526	0.000	0.526	0.053	0.053	0.474	0.789
	Specificity	0.591	1.000	0.727	1.000	0.955	0.727	0.955
	AUC	0.464	0.267	0.590	0.353	0.285	0.591	0.935
	Cutoff	2191.830	4777.510	17603.985	1003.700	869.660	180.000	0.721
	95% CI[a]	0.282-0.646	0.109-0.424	0.411-0.769	0.181-0.525	0.122-0.447	0.415-0.767	0.862-1.000
BM vs PD	Sensitivity	0.708	0.542	0.542	0.708	0.667	0.417	0.667
	Specificity	0.636	0.682	0.773	0.773	0.591	0.909	0.955
	AUC	0.650	0.533	0.614	0.808	0.596	0.606	0.871
	Cutoff	2352.855	7.100	17416.560	458.530	540.815	264.780	0.669
	95% CI[a]	0.489-0.810	0.364-0.703	0.447-0.780	0.685-0.931	0.428-0.763	0.440-0.772	0.770-0.972
BD vs HCs	Sensitivity	0.760	0.680	0.920	0.840	0.640	0.480	0.920
	Specificity	0.789	0.579	0.263	0.842	0.895	0.947	0.947
	AUC	0.815	0.636	0.514	0.886	0.753	0.672	0.975
	Cutoff	2746.785	5.420	13665.750	398.140	575.170	588.830	0.520
	95% CI[a]	0.688-0.941	0.470-0.801	0.326-0.701	0.785-0.987	0.607-0.899	0.512-0.832	0.937-1.000
BD vs PD	Sensitivity	0.625	0.917	0.542	0.708	0.750	0.417	0.958

	Specificity	0.789	0.526	0.684	0.947	0.789	0.789	0.895
	AUC	0.684	0.750	0.531	0.894	0.755	0.535	0.978
	Cutoff	2571.555	4.805	18037.950	461.330	521.180	264.780	0.482
	95% CI[a]	0.521-0.847	0.602-0.898	0.355-0.706	0.798-0.989	0.606-0.905	0.360-0.710	0.945-1.000
PD vs HCs	Sensitivity	0.720	0.000	0.920	0.600	0.320	0.480	0.640
	Specificity	0.583	1.000	0.333	0.542	0.917	0.833	0.917
	AUC	0.664	0.398	0.440	0.520	0.570	0.631	0.802
	Cutoff	2952.375	16.700	13649.475	507.510	940.830	658.200	0.592
	95% CI[a]	0.512-0.817	0.237-0.558	0.265-0.615	0.355-0.685	0.405-0.735	0.473-0.789	0.677-0.927

Notes: ×6: combination of tPA, PAI-1, BDNF, proBDNF, TrkB and p75NTR; [a]95% CI for AUC.

Abbreviations: AUC, area under the curve; SZ, schizophrenia; MDD, major depressive disorder; BM, bipolar mania; BD, bipolar depression; PD, panic disorder; HCs, healthy controls; BMI, body mass index; SAPS, Scale for Assessment Positive Symptom; HARS, Hamilton Anxiety Rating Scale; HDRS, 17-item Hamilton Depression Rating Scale; YMRS, Young Mania Rating Scale; PDSS, Panic Disorder Severity Scale; tPA, tissue plasminogen activator; PAI-1, plasminogen activator inhibitor-1; BDNF, brain-derived neurotrophic factor; pro-BDNF, precursor-BDNF; TrkB, tropomyosin-related kinase B; p75NTR, neurotrophin receptor p75.
