# Supplementary material for: Combined serum levels of multiple proteins in tPA-BDNF pathway may aid the diagnosis of five mental disorders
Source: Sci Rep. 2017 Jul 31;7:6871. doi: 10.1038/s41598-017-06832-6 (PMC5537244; doi:10.1038/s41598-017-06832-6)
Supplement: Supplementary file 3 — Supplementary Figure [file 41598_2017_6832_MOESM3_ESM.pdf]

Combined serum levels of multiple proteins in tPA-BDNF pathway may aid the diagnosis of five mental disorders

Suzhen Chen, MD[1,2], Haitang Jiang, MD[1,2], Yang Liu, BD[3], Zhenhua Hou, MD[1,2], Yingying Yue, MD[1,2], Yuqun Zhang, MD[1,2], Fuying Zhao, MD[1,2], Zhi Xu, PhD[1,2], Yinghui Li, MD[1,2], Xiaodong Mou, PhD[1,2], Lei Li, MD[1,2], Tianyu Wang, MD[1,2], Jingjing Zhao, BD[4], Chongyang Han, MD[4], Yuxiu Sui, PhD[4], Ming Wang, BD[5], Zhong Yang, BD[5], Yan Lu, BD[6], Yifeng Zhu, BD[6], Jianhua Li, MD[7], Xinhua Shen, MD[7], Fei Sun, BD[8], Qingsong Chen, BD[8], Huanxin Chen, PhD[9] & Yonggui Yuan, PhD[1,2*]

[1]Department of Psychosomatics and Psychiatry, ZhongDa Hospital, Medical School of Southeast University, Nanjing 210009, PR China.

[2]Institute of Psychosomatics, Medical School of Southeast University, Nanjing 210009, PR China.

[3]Institute of Neuropsychiatric, Brain Hospital, Nanjing Medical University, Nanjing 210029, PR China.

[4]Department of Psychiatry, Brain Hospital, Nanjing Medical University, Nanjing 210029, PR China.

[5] Department of Psychiatry, The Third People's Hospital of Changshu, Suzhou 215500, PR China.

[6]Department of Psychiatry, The Fourth People's Hospital of Zhangjiagang, Suzhou 215600, PR China.

[7]Department of Psychiatry, The Third People's Hospital of Huzhou, Huzhou 313000, PR China.

[8]Department of Psychiatry, The Second People's Hospital of Jingjiang, Taizhou 214500, PR China.

[9]Key Laboratory of Cognition and Personality, Ministry of Education; School of Psychology, Southwest University, Chongqing 400175, PR China.

*Corresponding author at: Department of Psychosomatics and Psychiatry, ZhongDa Hospital, Medical School of Southeast University, Nanjing 210009, PR China.

Tel./fax:+86 25 83285124; E-mail address: yygylh2000@sina.com (Y. Yuan).

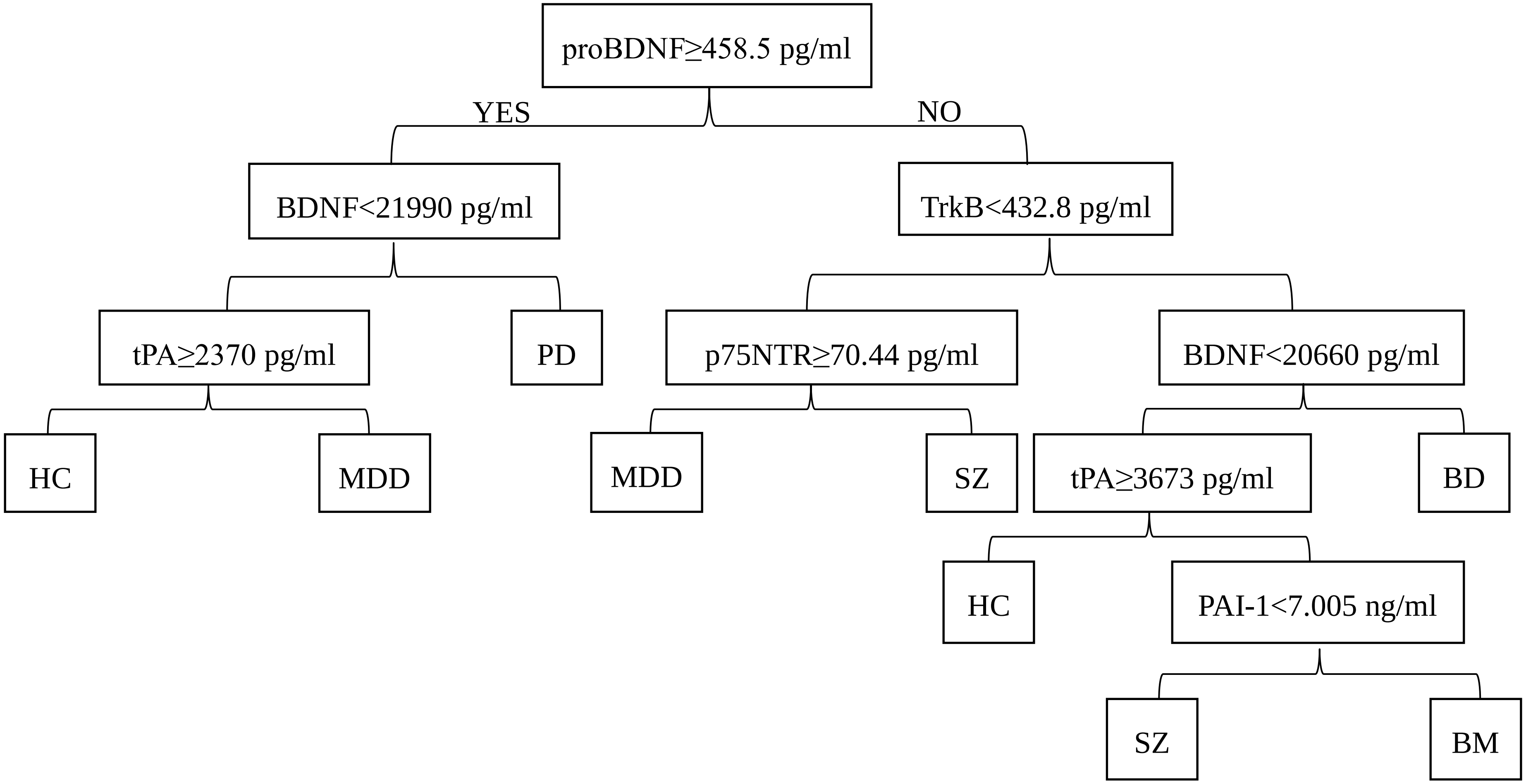
proBDNF≥458.5 pg/ml
YES
NO
BDNF<21990 pg/ml
TrkB<432.8 pg/ml
tPA≥2370 pg/ml
PD
p75NTR≥70.44 pg/ml
BDNF<20660 pg/ml
HC
MDD
MDD
SZ
tPA≥3673 pg/ml
BD
HC
PAI-1<7.005 ng/ml
SZ
BM

Supplementary Figure: The tree model for the diagnosis of various mental disorders.

Note: YES indicates the condition is satisfied; NO indicates the condition is not met.

Abbreviations: SZ, schizophrenia; MDD, major depressive disorder; BM, bipolar mania; BD, bipolar depression; PD, panic disorder; HCs, healthy controls; tPA, tissue plasminogen activator; PAI-1, plasminogen activator inhibitor-1; BDNF, brain-derived neurotrophic factor; proBDNF, precursor brain-derived neurotrophic factor; TrkB, tropomyosin-related kinase B; p75NTR, neurotrophin receptor p75.

The decision tree method is a powerful alternative to traditional linear models and has documented advantages for criteria generation. The decision tree model reveals the interactions between the variables and the outcomes, and the graphical outputs of the decision tree analyses could quantify and summarize the interactions visually. Thus, we used the rpart package in R to construct a tree model for the different diseases.

This model shows the decision tree with 7 internal nodes and 9 leaves. The divide criterion is indicated in each internal node. The proBDNF is the first factor to be observed, the index of proBNDF was defined as a cut-off value 458.5pg/ml. And then, the BNDF and TrkB assessment was placed at the second level, the tPA, p75NTR and BDNF at the third level, the tPA at the fourth level and the PAI-1 at the fifth level. Thus, the type of disease of the patients was assessed within 5 steps.
